# Phylogenetic and Phyletic Studies of Informational Genes in Genomes Highlight Existence of a 4^th^ Domain of Life Including Giant Viruses

**DOI:** 10.1371/journal.pone.0015530

**Published:** 2010-12-02

**Authors:** Mickaël Boyer, Mohammed-Amine Madoui, Gregory Gimenez, Bernard La Scola, Didier Raoult

**Affiliations:** URMITE, Centre National de la Recherche Scientifique UMR IRD 6236, Faculté de Médecine, Université de la Méditerranée, Marseille, France; Institute of Infectious Disease and Molecular Medicine, South Africa

## Abstract

The discovery of Mimivirus, with its very large genome content, made it possible to identify genes common to the three domains of life (*Eukarya*, *Bacteria* and *Archaea*) and to generate controversial phylogenomic trees congruent with that of ribosomal genes, branching Mimivirus at its root. Here we used sequences from metagenomic databases, Marseillevirus and three new viruses extending the *Mimiviridae* family to generate the phylogenetic trees of eight proteins involved in different steps of DNA processing. Compared to the three ribosomal defined domains, we report a single common origin for Nucleocytoplasmic Large DNA Viruses (NCLDV), DNA processing genes rooted between *Archaea* and *Eukarya*, with a topology congruent with that of the ribosomal tree. As for translation, we found in our new viruses, together with Mimivirus, five proteins rooted deeply in the eukaryotic clade. In addition, comparison of informational genes repertoire based on phyletic pattern analysis supports existence of a clade containing NCLDVs clearly distinct from that of *Eukarya*, *Bacteria* and *Archaea*. We hypothesize that the core genome of NCLDV is as ancient as the three currently accepted domains of life.

## Introduction

Molecular sequence analyses have allowed to partially identify the origins of genes. A putative tree of life based on ribosomal analysis was postulated that includes three domains: *Eukarya*, *Bacteria* and *Archaea*. Viruses are excluded from this classification system due to a lack of evidence that they possess a core of genes and because they lack ribosomes [1]. The double jelly-roll motif that can be found in the capsids of viruses infecting *Archaea*, *Eukarya*, or *Bacteria*, however, is proposed as evidence for an ancestry of some viruses [2], [3]. The description of the giant Mimivirus [4] that share similar features with cells has recently generated much debate about the nature of viruses in the living world [5]–. In our study, we identify a common set of proteins present in all forms of life including cellular organisms and viruses, particularly giant viruses, and we used this set of viral and cellular proteins to perform phylogenetic reconstructions.

## Results

**Figure 1:**
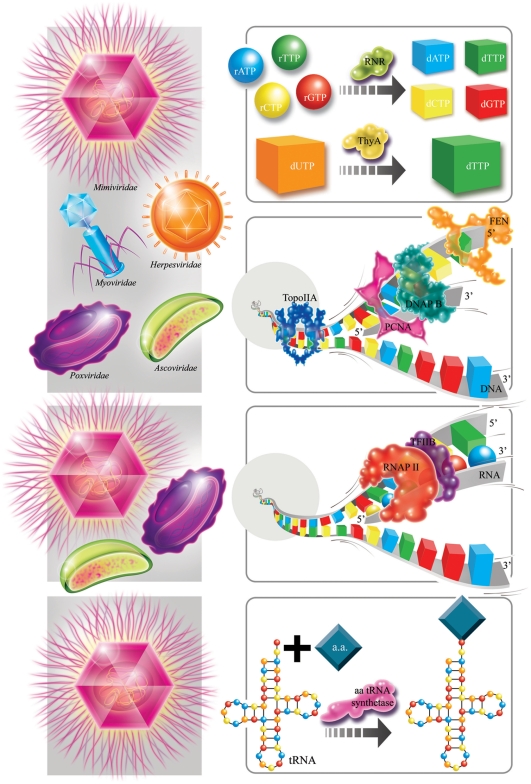
NCLDV proteins involved in DNA, RNA and protein biosynthesis. The frames on the right represent DNA processing steps with related enzymes found in NCLDVs, and the frames on the left with grey backgrounds show viruses that have the corresponding enzymes. The first frame from the top right corresponds to the dNTP biosynthesis catalyzed by RNR and ThyA. The second frame shows four enzymes involved in DNA replication: DNAP B, TopoIIA, PCNA and FEN. Proteins involved in these two DNA biosynthesis steps were found in diverse large DNA viruses, such as NCLDVs, *Herpesviridae* and *Myoviridae*. The third frame represents the DNA transcription step, in which the RNAP II and the transcription factor TFIIB are involved; viral homologs of these proteins are only found in NCLDVs. The last frame shows *Mimiviridae* amino-acyl tRNA synthetases (aa tRNA synthetase) involved in mRNA translation. Interestingly, viral lineages were consistently less well represented from nucleotide to protein biosynthesis, with the exception of NCLDVs, which were represented in each step. a.a., amino acid.

In modern cells, biosynthesis of DNA precursors (dNTPs) is achieved in two steps by two essential enzymes (Figure 1): ribonucleotide reductase (RNR) and thymidylate synthase (TdS) [8]. First, reduction of RNA precursors (rNTP) into dNTP is catalyzed by RNR, then deoxythymidine 5′-monophosphate (dTMP) is produced from deoxyuridine 5′-monophosphate (dUMP) by thymidylate synthase. These two key enzymes are thought to have been involved in the transition from ancient RNA to the DNA world [9]. They have been identified in cells and in nucleocytoplasmic large DNA viruses (NCLDVs) and used for phylogenetic reconstructions. RNR proteins have been classified into three classes [10]; we focus on classes I and II as class III proteins share very little similarity with the other two classes [11]. The RNR phylogeny (Figure S1) shows prokaryotic and eukaryotic clades and supports the emergence of RNR of three NCLDV families separately from the eukaryotes. Two families of non-homologous TdS, ThyA and ThyX, have been identified in living organisms, but the thymidylate synthases reported in NCLDVs are mostly of the ThyA type [12]. The phylogenetic reconstruction of the ThyA type (Figure S2) showed two clusters, one that includes bacteria, bacteriophages and *Archaea*, and the other that includes eukaryotes and their viruses. In the second group, ThyA in all NCLDV lineages was scattered within the eukaryotic clade, except for ThyA from Mimivirus and Marseillevirus, which emerge on a separate clade. Phylogenetic trees inferred for these two proteins also show NCLDV branches within eukaryotic clades. We assumed, as others have [13], that multiple gene exchanges or non-orthologous gene displacements have occurred during the extensive evolution between eukaryotes and viruses, thus blurring the topology.

Four key proteins are involved in DNA replication and repair. Three enzymes, including the DNA polymerase family B (DNAP B), the topoisomerase II A (TopoIIA), the Flap endonuclease (FEN), and the processing factor Proliferating Cell Nuclear Antigen (PCNA) (Figure 1) are largely distributed in NCLDVs [13] and are therefore used for phylogenetic reconstruction. The DNAP B tree (Figure S3) was built from archaeal, bacterial, viral and eukaryotic α, δ and ζ protein sequences' alignment. Sequences from NCLDVs and *Herpesviridae* were sister clades rooted between the δ and ζ eukaryotic subfamilies. The TopoIIA tree (Figure S4) was obtained from archaeal, bacterial, eukaryotic and NCLDVs' sequences' alignment. The TopoIIA phylogenetic reconstruction showed eukaryotic monophyly, whereas NCLDVs are not monophyletic but emerged as a bush between the eukaryotic and prokaryotic clades. PCNA and FEN were not found in *Bacteria* and their respective phylogenetic trees (Figures S5 and S6, respectively), supporting distinct *Archaea*, eukaryote and NCLDV monophylies. Therefore, these four protein phylogenies support, with a high degree of confidence, the existence of a viral clade with ancestral DNA replication machinery branching separately from *Eukarya* and *Archaea*.

**Figure 2:**
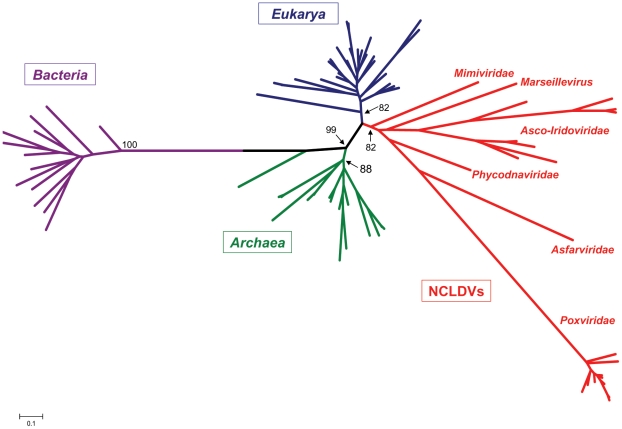
Phylogenetic tree of the RNA polymerase II beta subunit. The ML tree of RNAP II was inferred from a cured alignment of 80 sequences from the six supergroups of *Eukarya* (blue), *Bacteria* (purple), *Archaea* (green) and NCLDVs (red). The tree is unrooted, and values near branches are SH-like local supports computed by the FastTree program and are used as confidence values of tree branches. Scale bar represents the number of estimated changes per position for a unit of branch length.

**Figure 3:**
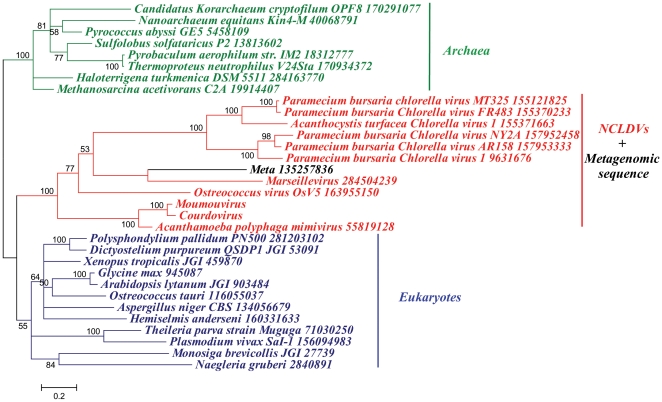
Phylogenetic tree of the Transcription factor II B (TFIIB). The TFIIB phylogenetic tree is inferred with Bayesian approach from a cured alignment of 32 sequences (155 conserved positions) from the *Eukarya* (blue), *Archaea* (green), NCLDVs (red), and metagenomic databases (black). Bayesian posterior probabilities are mentioned near branches as a percentage and are used as confidence values of tree branches. Scale bar represents the number of estimated changes per position for a unit of branch length.

In addition, we investigated the phylogenetic reconstruction of two proteins involved in transcription processing: the DNA-dependant RNA polymerase II (RNAP II) is a ubiquitous enzyme, and its phylogenetic tree reveals a clade containing NCLDVs clearly distinct from that of *Eukarya*, *Bacteria* and *Archaea* (Figure 2 and Figure S7). Transcription factor II B (TFIIB), absent in bacteria, is a general transcription factor that makes up the RNA polymerase II pre-initiation complex. Its phylogenetic analysis displayed similar results (Figure 3), with the exception that bacteria are absent. These results lead to the conclusion that highly conserved proteins involved in the RNA biosynthesis in NCLDVs have emerged in a clade as ancient as those of eukaryotes and *Archaea*.

The Mimivirus genome contains several genes encoding proteins involved in DNA translation [4]. We investigated a phylogenetic reconstruction for five proteins involved in translation, including four amino-acyl tRNA synthetases (Figures S8, S9, S10, S11) and the putative elongation factor EF-1 (Figure S12), which are also found in the draft genomes of three newly described members of the *Mimiviridae* family (Terravirus, Courdovirus and Moumouvirus) [14]. Arginyl-tRNA synthetase and cysteine-tRNA synthetase phylogenetic trees (Figures S8 and S10) show blurred topologies of *Mimiviridae* branching inside the eukaryotes clade, but the topology of the methyonyl-tRNA synthetase tree (Figure S11), which contains three distinct eukaryotic clades, supports the existence of a *Mimiviridae* clade branching before the eukaryotic clade containing *Amoebozoa* and opisthokonts. The topology of the tyrosyl-tRNA synthetase tree supports the hypothesis of a transfer from an ancient viral clade to an amoeba [15], [16]. It was previously suggested that the amino-acyl tRNA synthetases are more commonly exchanged genes [17], and the amino-acyl tRNA synthetase trees, including that of *Mimiviridae*, support this hypothesis and show that viruses are also included in the confused evolutionary scenarios of these proteins. Proteins related to EF-1 are divided into different subfamilies according to their functional domains [18]. That of *Mimiviridae* displays the GTP-binding protein domain (GTPBP1) found in a protein family divergent from the bona fide EF-1 family [19]. The phylogenetic tree (Figure S12) exhibited three large clades corresponding to the archaeal and eukaryotic translation protein families related to EF-1, one clade corresponding to the divergent bacterial elongation factor EF-TU, and another corresponding to GTPBP1 proteins. A phylogenetic tree supported the emergence of the *Mimiviridae* elongation factor inside the GTPBP1 eukaryote clade. In conclusion, the phylogenetic trees based on translational proteins suggest that *Mimiviridae* acquired some of the genes at the root of the eukaryote clade.

**Figure 4:**
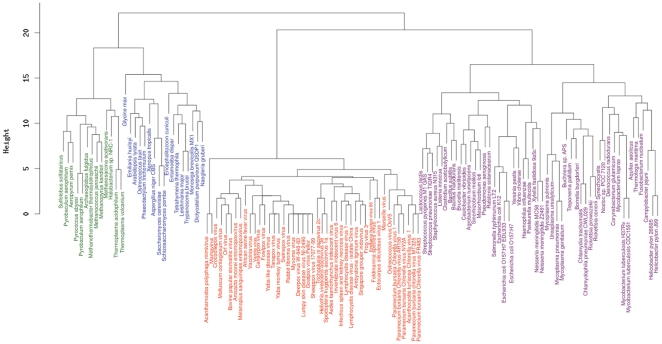
Hierarchical clustering of *Eukarya* (blue), *Bacteria* (purple), *Archaea* (green) and NCLDVs (red) by phyletic pattern. The phyletic patterns of the putative orthologous sets of informational genes indicating the presence/absence of the respective gene in each cellular organisms and virus were used for the construction of the dendogram tree.

As DNA processing genes appear to be the most conserved genes between cellular organisms and viruses, we further constructed a different type of tree, known as phyletic pattern [20], that is based on the comparison of the gene repertoire involved in the information storage and processing. A dendogram tree (Figure 4) was constructed from the presence/absence of the respective genes of each virus and cellular organisms in database of Clusters of Orthologous Groups (COGs) related to the DNA processing functions. The topology of this gene content tree supports the existence of a clade containing NCLDVs clearly distinct from that of *Eukarya*, *Bacteria* and *Archaea*. In accordance with the phylogenetic tree based on NCLDV core genes [13], viruses of the same family roughly grouped together inside the NCLDV clade. This tree based on informational gene repertoire is compatible with the above phylogenetic trees showing clearly four distinct clades and with that of the ribosomal phylogenetic trees (excluding viruses).

## Discussion

Our results attest to the existence of a single and ancestral source of genes allowing for the biosynthesis of DNA in large DNA viruses. Based on the study of these genes, we speculate that they appeared simultaneously with or just after the emergence of the modern eukaryote lineages. This topology may be biased by the fact that the current eukaryotes, arising from the fusion of a proto-eukaryote with an alpha-proteobacteria, emerged a billion years ago. This was followed by a bottleneck and the disappearance of all proto-eukaryotes that did not successfully form a symbiotic fusion with bacteria [21]. Therefore, there are no traces of the viruses of these proto-eukaryotes. This might explain the branching of some NCLDVs inside the eukaryote clade. Finally, an analysis of the current genealogy of organisms showed that the group of genes associated with DNA synthesis has a common source that is conserved in three or four current domains of organisms (including viruses) and had a topology comparable to that of ribosomal phylogenetic trees (excluding viruses). Finally, if we consider these nine proteins, five were present in the three canonical domains and NCLDVs; the other four trees included only three clades: the ribosomal tree and three containing NCLDVs and two domains (PCNA, FEN, and TFIIB). We conclude that trees based on ribosomal proteins are not sufficient to represent all forms of life as they do not include viruses.

In addition, as previously suggested [22], it is possible that the ancestral cellular DNA machinery came from DNA viruses, which would have thus in turn provided genes allowing DNA replication. This work also confirms and extends the seminal work of Iyers *et al.*, who identified for the first time a core genome of the NCLDV with nine genes common to all species of this group of viruses [23]. Interestingly, the name NCLDV does not reflect the striking complexity of this group of viruses that may contain both DNA and RNA [4]. Finally in this study, we found that the informational gene repertoires, irrespective of their individual source, are also evidencing four major clades.

**Figure 5:**
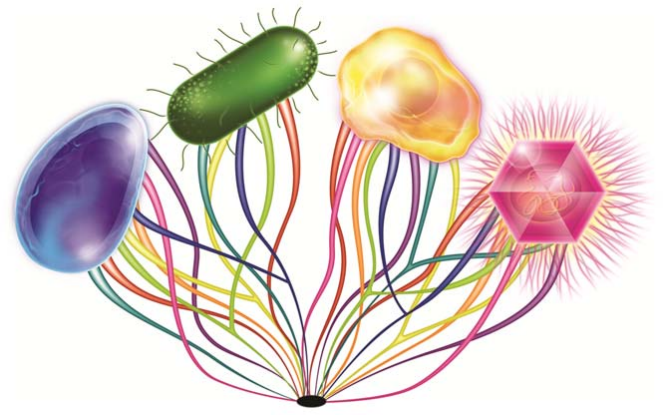
Scenario of NCLDVs emerging from the rhizome of life with roots appearing at the very beginning of life. This figure represents the living species in the four small pictures according to the current classification of organisms: eukaryotes (represented by yellow cell), bacteria (represented by green cell), *Archaea* (represented by blue cell) and viruses (represented by magenta colored Mimivirus). An organism classification based on ribosomal proteins allows the discrimination of cellular organisms, but viruses are excluded de facto from historical scenarios for life evolution. Our study shows that eight proteins involved in DNA processing and present both in viruses, particularly in NCLDVs, and in two or three other domains of life could be used for phylogenetic tree reconstruction, displaying schematic topologies for each protein represented by lines colored in light green for RNR, in yellow for ThyA, in blue-green for DNAP B, in blue for TopoIIA, in magenta for PCNA, in orange for FEN, in red for RNAP II and in purple for TFIIB. Topology representations were similar to that based on ribosomes (for RNAP II, FEN, PCNA and TFIIB proteins) and/or with bifurcations (for ThyA, RNR, DNAP B and Topo IIA proteins) representing probable lateral gene transfer. Thus, this figure illustrates that REOs and CEOs (bona fide viruses) share a common set of genes involved in DNA processing that evolved from a common ancestral source of genes. The phenomenon of long-branch attraction makes the identification of the exact deep rooting of each analyzed gene difficult and is therefore hidden by a black mark.

Because the set of genes associated with the DNA machinery is found in both viruses and cells, the origin of this gene set may be questionable, and some authors have suggested that large DNA viruses might have contributed to the formation of the nucleus of eukaryotes [24], [25]. In this scenario, current forms of life would result from the fusion of the world of RNA and proteins (including ribosomes) with that of DNA; RNA and protein molecules have been preserved in modern cells (*Archaea*, *Bacteria* and *Eukarya*), whereas some viruses (i.e., *Mimiviridae*) containing both RNA and DNA have conserved translation-associated proteins such as tRNA synthetases, which are probably remnants of an ancestral translation apparatus. In any case, we speculate that DNA machinery is a clear remnant of a biological “Big Bang” generating at least two distinct forms of life: DNA-based and DNA-RNA-ribosome-based. Since this ancestral time, two forms of life have co-developed [6]; cells that are ribosome-encoding organisms (REOs), diversified in three domains (*Archaea*, *Bacteria*, and *Eukarya*), and capsid-encoding organisms (CEOs) (bona fide viruses, parasites of the REOs). Accepting this ancestry allows us to reinterpret the origin of two-tailed virus families (i.e., *Myoviridae* and *Siphoviridae*) infecting both bacteria and *Archaea*. One can suggest that they derive from ancestral viruses infecting the REO ancestor rather than being transferred from one domain to another [26]. This makes sense if one considers that *Siphoviridae* appear to be the most common viral family on Earth [27]. Finally, several previous studies focusing on the common genetic features in viruses from *Archaea* and from eukaryotes [28], [29] suggest that there is a conserved endonuclease structure in influenza virus and in the endonucleases of *Archaea* and bacteria [30]. Evidence that the picorna-like superfamily of RNA viruses originated before the radiation of eukaryotes [31] and a phylogenetic tree of the guanylyl transferase domain branching poxviruses at the origin of eukaryotes [25] might also be reinterpreted in this light and are congruent with the hypothesis of CEO ancestry. Based on these data, we propose a scenario in which NCLDV emerged from the rhizome of life [32] with roots arising at the very beginning of life (Figure 5).

## Materials and Methods

### Groups of homologs common to the three domains of life and NCLDVs

Selected proteomes of organisms representing major phyla of the three domains of life (*Eukarya*, *Archaea* and *Bacteria*) were downloaded from NCBI and JGI. BLASTP [33] searches were performed with this set of proteins to identify conserved proteins in the complete proteomes that are available for the six NCLDV families (*Asfarviridae*, *Asco*-*Iridoviridae*, *Phycodnaviridae*, *Poxviridae*, Marseillevirus and *Mimiviridae*) and the draft proteomes of three new *Mimiviridae* [14]. Groups of homologs were built using the criteria that sequences were homologous if the Best Blast Hit (BBH) showed an alignment length of over 70 amino acids and with a percent identity over 20%. Four proteins matching these criteria that were also present in at least the three domains of life were identified: thymidylate synthase, RNR, DNAP B, TopoIIA (also called gyrase in bacteria), and RNAP II. The FEN, the PCNA and the TFIIB were found only in eukaryotes and *Archaea*. Aminoacyl-ARNt synthetases and EF-1-like proteins were only identified in *Mimiviridae* and the three domains of life. For this set of proteins, homologs among all the viral proteomes together with homologs with the largest coverage relating to our inquiry in environmental sequence data (env_nr) were detected using BLASTP. The Courdovirus protein sequences have been deposited in GenBank under the following accession numbers: HQ223093 for cysteinyl-tRNA_synthetase, HQ223094 for tyrosyl-tRNA_synthetase, HQ223095 for methionyl-tRNA_synthetase, HQ223096 for arginyl-tRNA_synthetase, HQ223097 for GTPB1_like, HQ404368 for TFIIB. The Terravirus protein sequences have been deposited in GenBank under the following accession numbers: HQ223098 for cysteinyl-tRNA_synthetase, HQ223099 for tyrosyl-tRNA_synthetase, HQ223100 for methionyl-tRNA_synthetase, HQ223101 for arginyl-tRNA_synthetase, HQ223102 for GTPB1_like. The Moumouvirus protein sequences have been deposited in GenBank under the following accession numbers: HQ223103 for cysteinyl-tRNA_synthetase, HQ223104 for tyrosyl-tRNA_synthetase, HQ223105 for GTPB1_like, HQ404369 for TFIIB.

### Multiple sequence alignments and phylogenetic reconstruction

T-Coffee [34] or Muscle [35] was used to construct multiple alignments, and conserved blocks were selected using Gblocks [36]. The corrected alignments were then used for maximum likelihood (ML) and Bayesian inference (BI). ML phylogeny inference was constructed using FastTree [37], with the JTT+CAT substitution model. Values near branches are SH-like local supports computed by the FastTree program and are used as confidence values of tree branches. For the Bayesian approach, the phylogeny was performed using MrBayes[38]; the WAG matrix was used, and model parameters (gamma shape and proportion invariant) were allowed to vary through the Markov Chain Monte Carlo Chain (MCMC). Four MCMC chains were run for 1 million generations and sampled every 100th generation. The first 100,000 trees were discarded, and the sumt command of MrBayes was used to compute clade posterior probabilities. Trees were displayed using MEGA 4 [39].

### Construction of dendrogram gene content tree from phyletic patterns

Comparison of informational genes repertoire was performed using a phyletic pattern indicating the presence/absence of the respective informational genes of all species present in the corresponding functional categories of Clusters of Orthologous Group (COGs) based on 66 genomes [40], [41]. Only COGs functional categories related to Information storage and processing ([J, A, K, L and B] COG categories) and Nucleotide transport and metabolism ([F] COG category) were used to build a matrix by assigning as a “1” if there is at least one ortholog contained in a genome and a “0” if not. As eukaryotes are weakly represented in initial COG database, we extended the initial matrix by assigning a COG functional category to proteins of 14 added eukaryotic proteomes using BLASTP against COG database (e-value <10-3). We also incremented the matrix of phyletic pattern with NCLDVs species by assigning NCLDVs proteins in corresponding COGs, which is performed by linking functional categories of NCVOGs database [13] with those of COGs using the same parameters. The euclidian distance matrix was computed from the 0/1 matrix and then a dendogram tree was built from hierarchical clustering using in house R language.

## Supporting Information

Figure S1 **Bayesian phylogenetic tree of RNR (31 sequences, 166 positions).** GI or JGI numbers are listed next to the corresponding taxonomic name of each cellular organism and virus. A color code was used to represent taxonomic groups, *Bacteria* in purple, *Archaea* in green, *Eukarya* in blue, NCLDVs in red, other viruses and phages in pink and environmental sequences in black. Numbers at nodes are Bayesian posterior probabilities. Scale bar represents the number of estimated changes per position for a unit of branch length.
(PDF)

Figure S2 **Bayesian phylogenetic tree of ThyA (44 sequences, 159 positions).** Detailed legend is the same as in Figure S1.
(PDF)

Figure S3 **Bayesian phylogenetic tree of DNAP B (62 sequences, 139 positions).** Detailed legend is the same as in Figure S1.
(PPT)

Figure S4 **Bayesian phylogenetic tree of TopoIIA (48 sequences, 140 positions).** Detailed legend is the same as in Figure S1.
(PPT)

Figure S5 **Bayesian phylogenetic tree of PCNA (40 sequences, 174 positions).** Detailed legend is the same as in Figure S1.
(PPT)

Figure S6 **Bayesian phylogenetic tree of FEN (37 sequences, 304 positions).** Detailed legend is the same as in Figure S1.
(PPT)

Figure S7 **Bayesian phylogenetic tree of RNAP II (80 sequences, 272 positions).** Detailed legend is the same as in Figure S1.
(PPT)

Figure S8 **Maximum-likelihood phylogenetic tree of arginyl-tRNA synthetase (32 sequences, 123 positions).** Detailed legend is the same as in Figure S1 except that numbers at nodes are SH-like local supports.
(PPT)

Figure S9 **Maximum-likelihood phylogenetic tree of tyrosyl-tRNA synthetase (33 sequences, 201 positions).** Detailed legend is the same as in Figure S1 except that numbers at nodes are SH-like local supports.
(PPT)

Figure S10 **Maximum-likelihood phylogenetic tree of cysteine-tRNA synthetase (38 sequences, 156 positions).** Detailed legend is the same as in Figure S1 except that numbers at nodes are SH-like local supports.
(PPT)

Figure S11 **Maximum-likelihood phylogenetic tree of methyonyl-tRNA synthetase (51 sequences, 204 positions).** Detailed legend is the same as in Figure S1 except that numbers at nodes are SH-like local supports.
(PDF)

Figure S12 **Bayesian phylogenetic tree of EF-1 like phylogenetic tree (65 sequences, 157 positions).** Detailed legend is the same as in Figure S1.
(PPT)
