# Supplementary figures and images for: Phylogenetic and Phyletic Studies of Informational Genes in Genomes Highlight Existence of a 4th Domain of Life Including Giant Viruses
Source: PLoS One. 2010 Dec 2;5(12):e15530. doi: 10.1371/journal.pone.0015530 (PMC2996410; doi:10.1371/journal.pone.0015530)

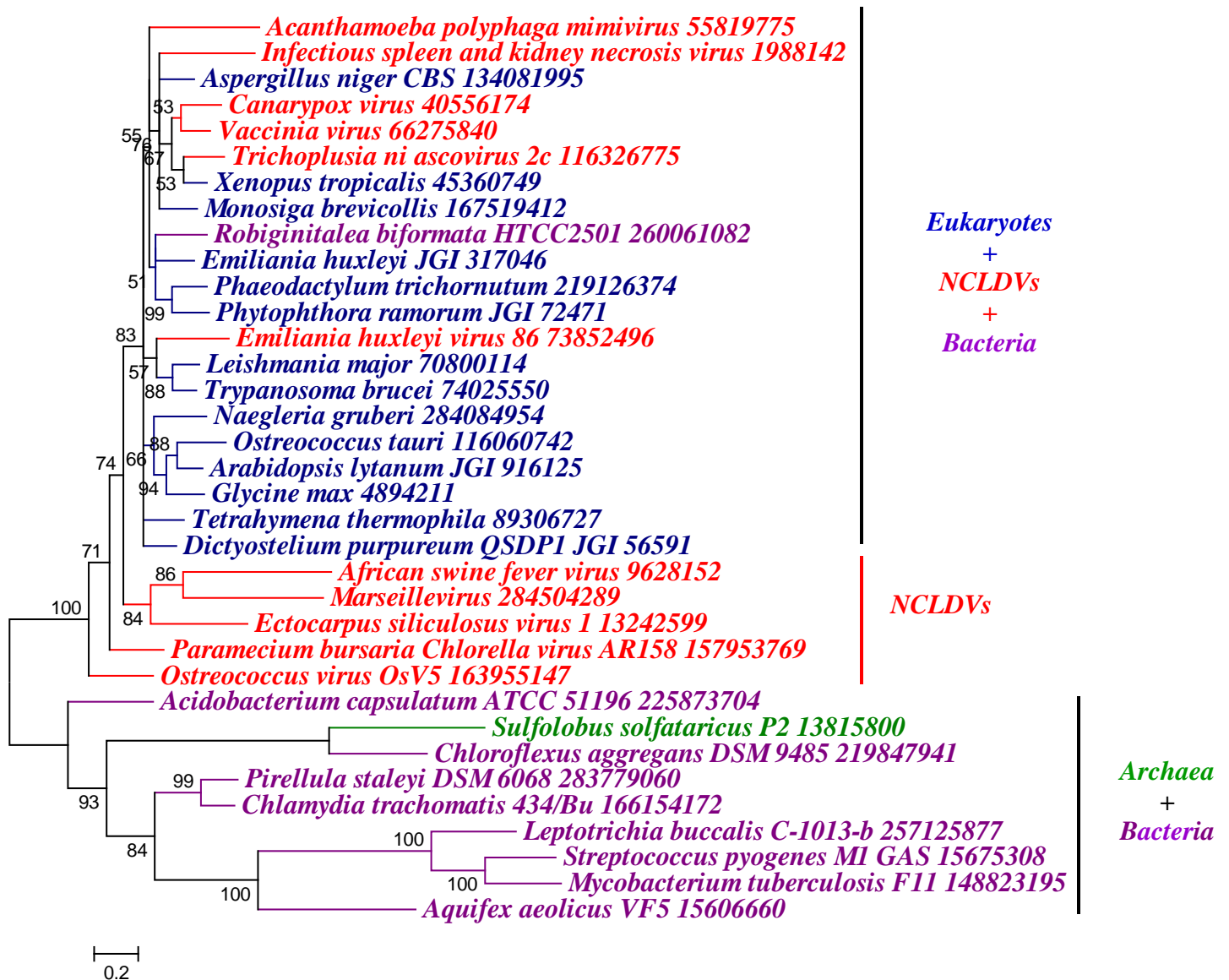

**Figure S1**

Supplement: Figure S1 — Bayesian phylogenetic tree of RNR (31 sequences, 166 positions). GI or JGI numbers are listed next to the corresponding taxonomic name of each cellular organism and virus. A color code was used to represent taxonomic groups, Bacteria in purple, Archaea in green, Eukarya in blue, NCLDVs in red, other viruses and phages in pink and environmental sequences in black. Numbers at nodes are Bayesian posterior probabilities. Scale bar represents the number of estimated changes per position for a unit of branch length. (PDF) [file pone.0015530.s001.pdf]

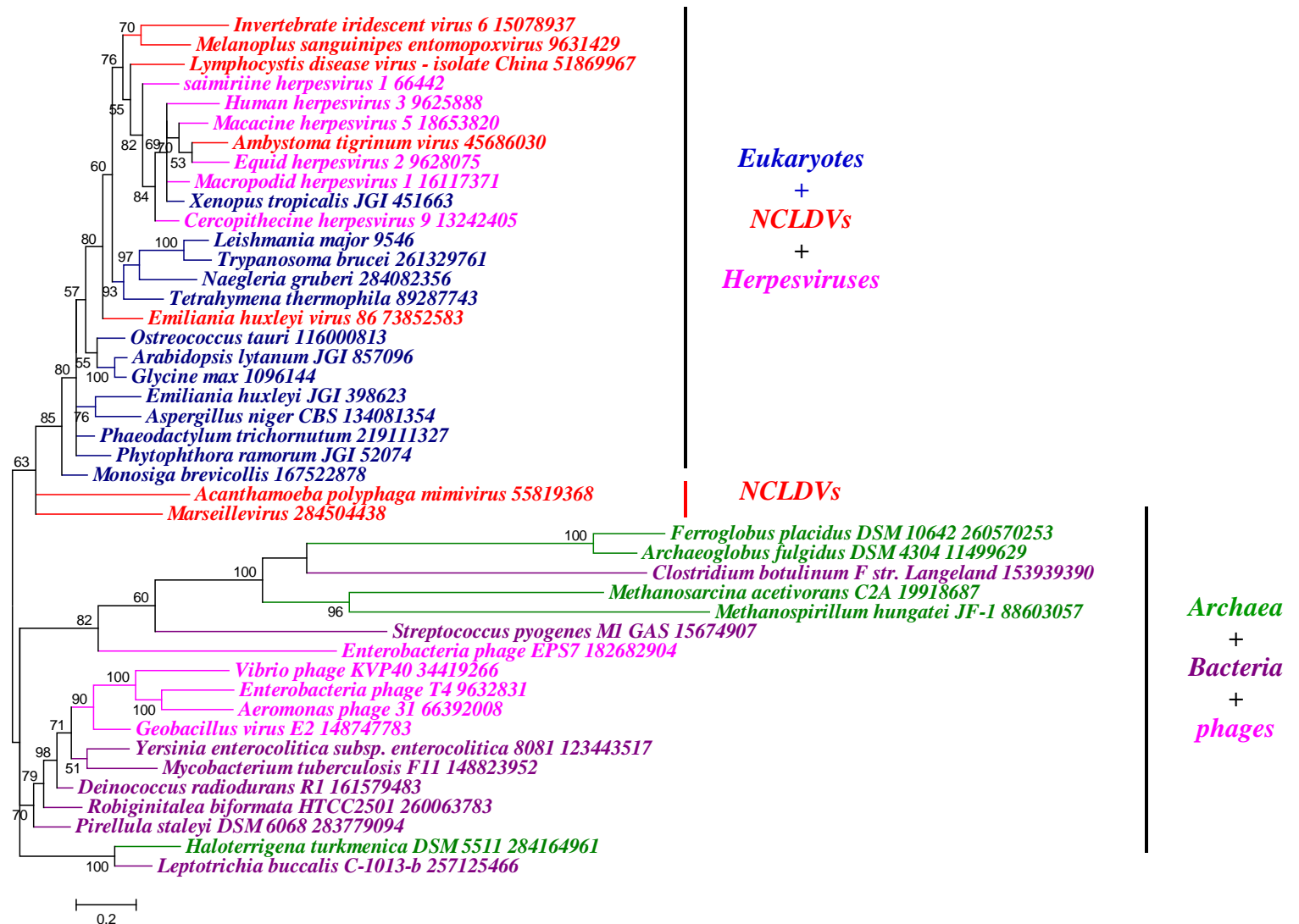

**Figure S2**

Supplement: Figure S2 — Bayesian phylogenetic tree of ThyA (44 sequences, 159 positions). Detailed legend is the same as in Figure S1. (PDF) [file pone.0015530.s002.pdf]

## Slide 1
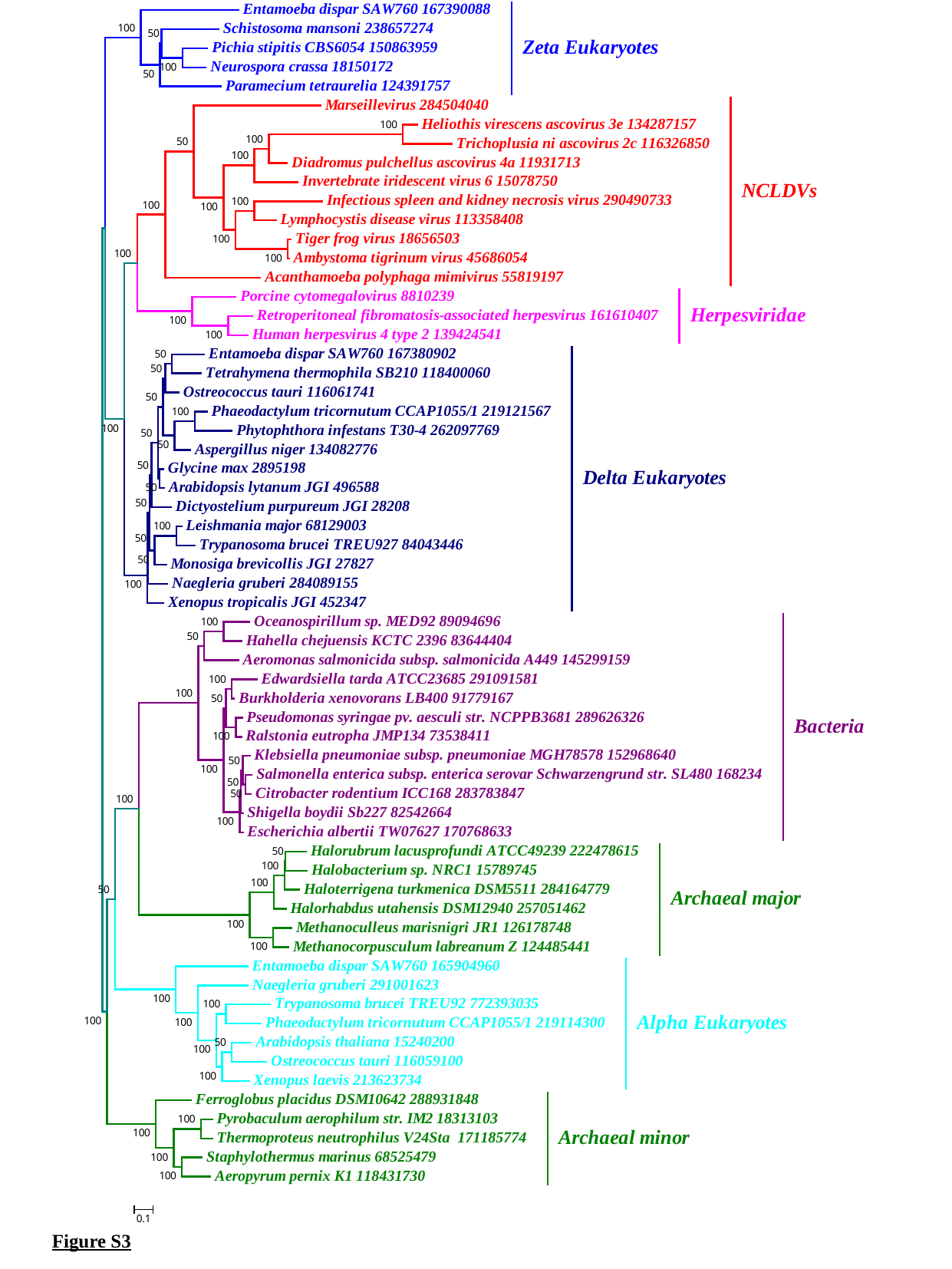

Figure S3

Supplement: Figure S3 — Bayesian phylogenetic tree of DNAP B (62 sequences, 139 positions). Detailed legend is the same as in Figure S1. (PPT) [file pone.0015530.s003.ppt]

## Slide 1
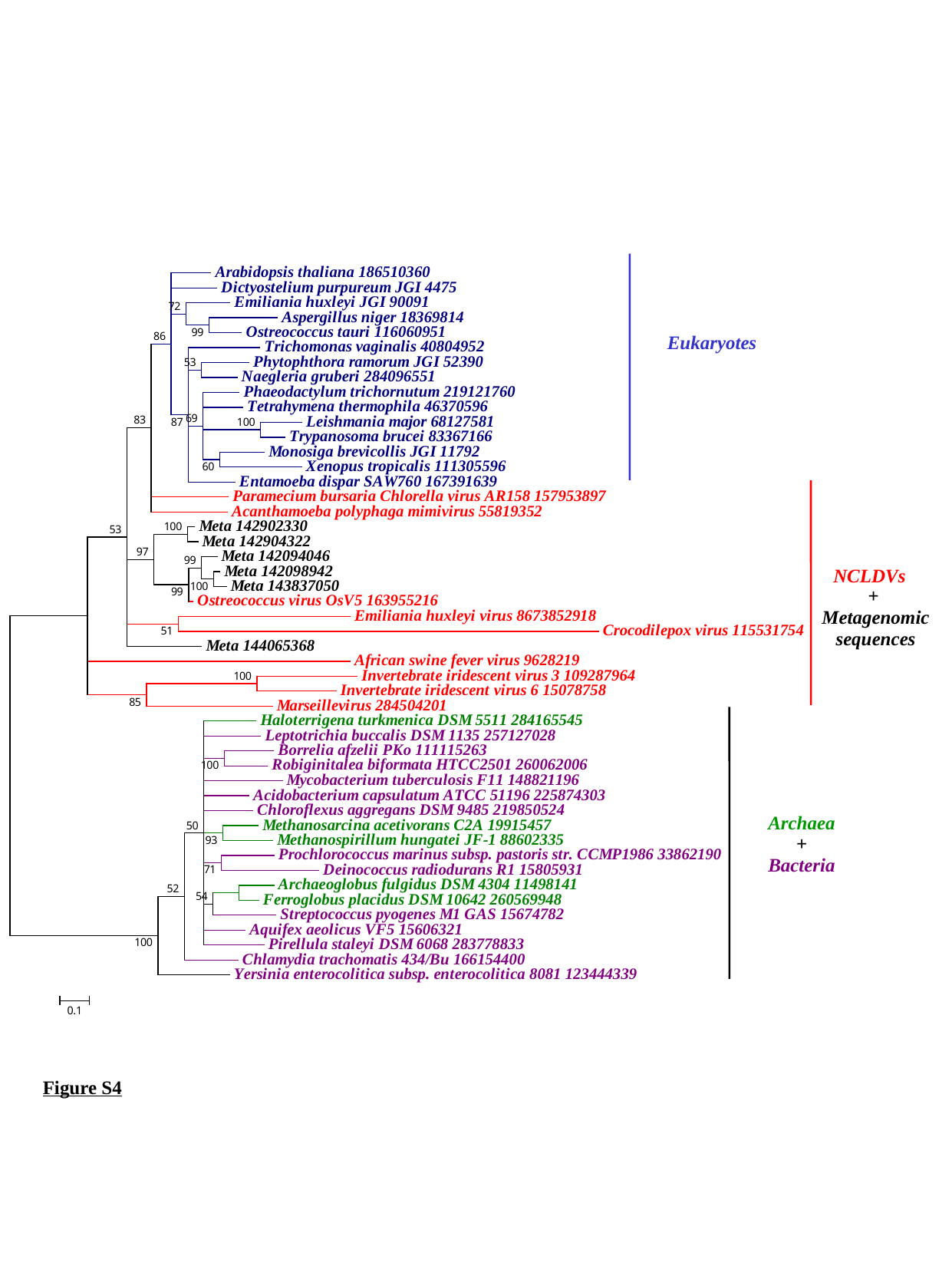

Eukaryotes
NCLDVs
+
Metagenomic sequences
Archaea
+
Bacteria
Figure S4

Supplement: Figure S4 — Bayesian phylogenetic tree of TopoIIA (48 sequences, 140 positions). Detailed legend is the same as in Figure S1. (PPT) [file pone.0015530.s004.ppt]

## Slide 1
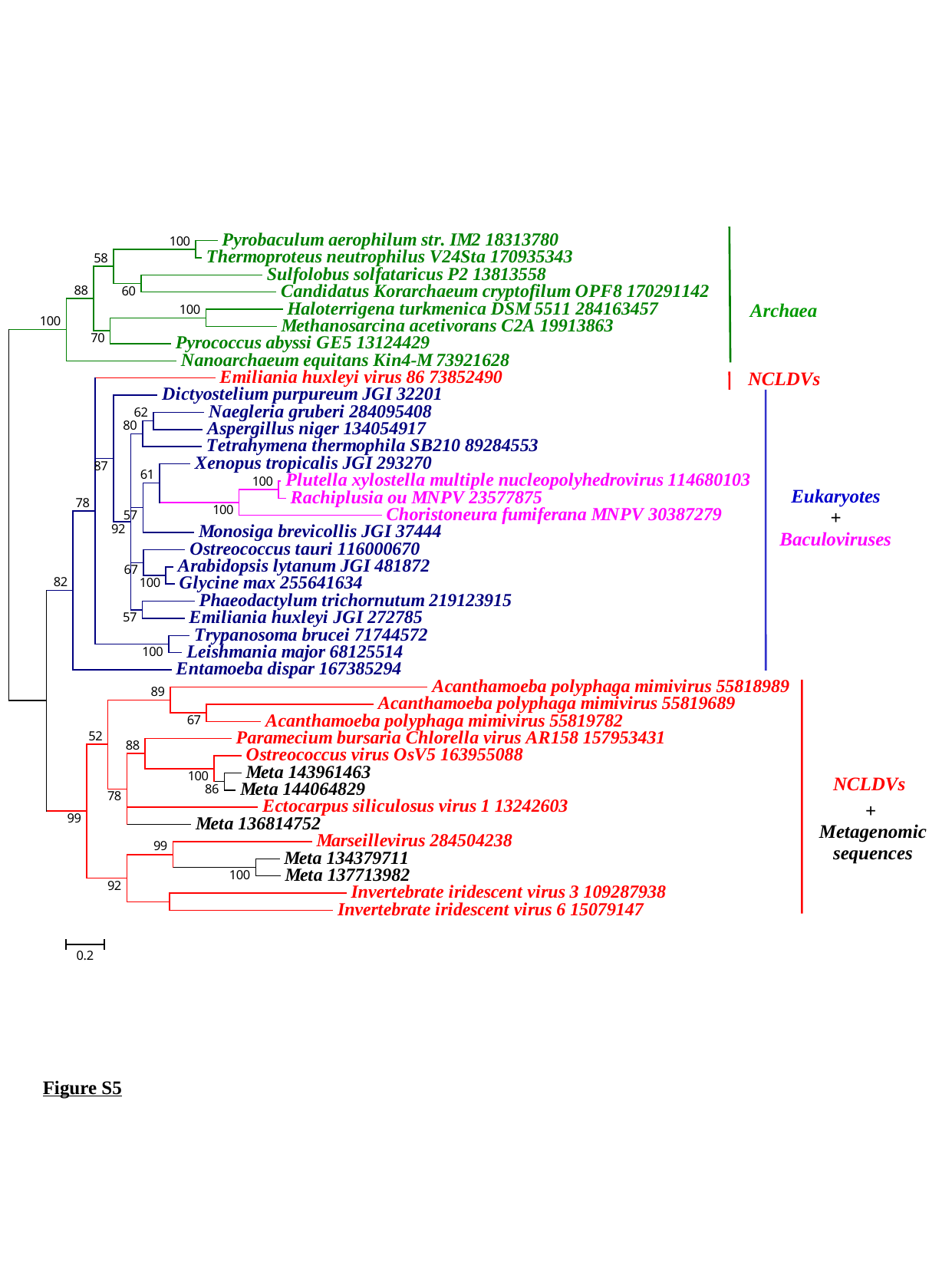

Archaea
NCLDVs
Eukaryotes
+
Baculoviruses
NCLDVs
+
Metagenomic sequences
Figure S5

Supplement: Figure S5 — Bayesian phylogenetic tree of PCNA (40 sequences, 174 positions). Detailed legend is the same as in Figure S1. (PPT) [file pone.0015530.s005.ppt]

## Slide 1
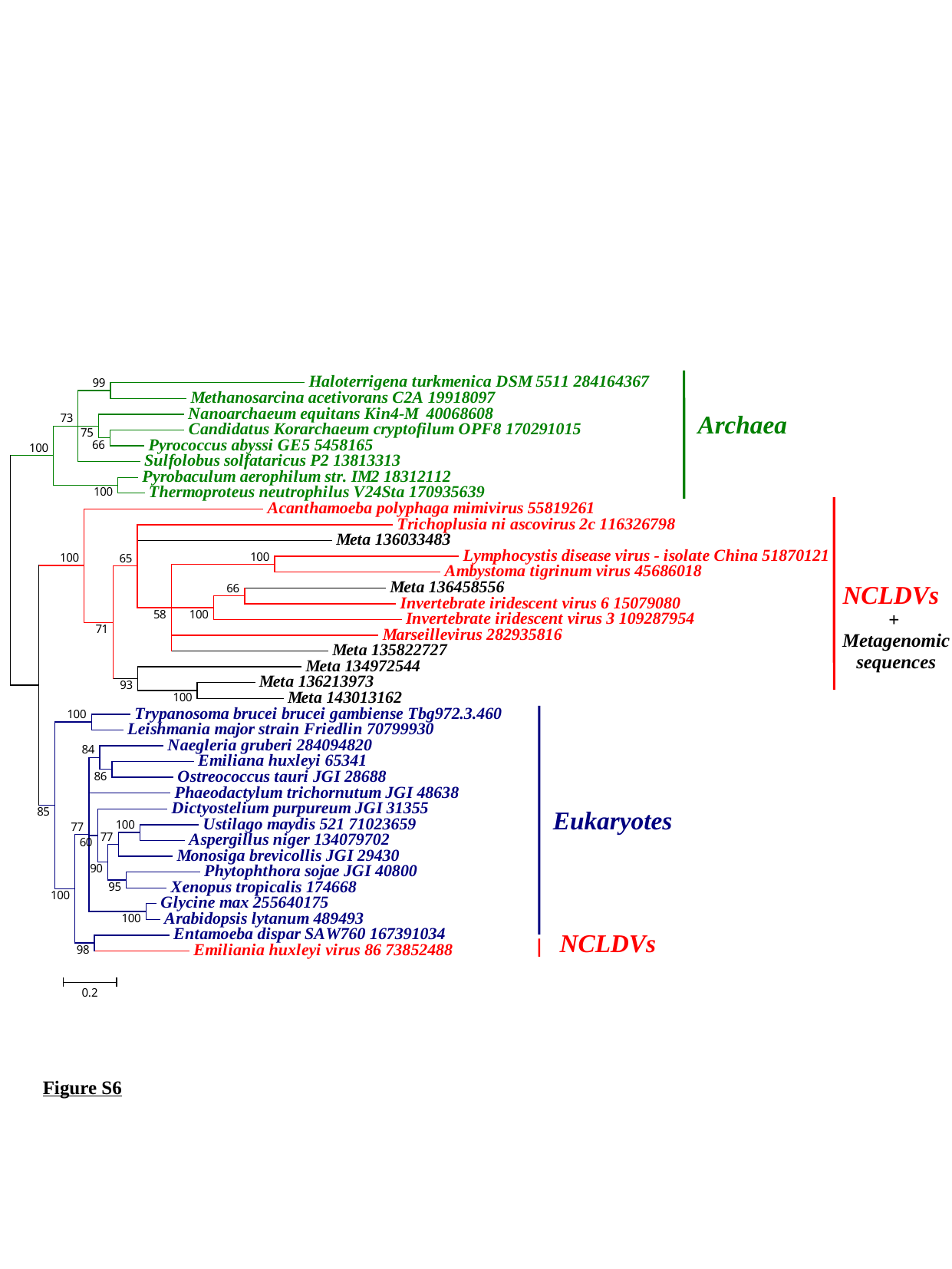

Archaea
NCLDVs
+
Metagenomic sequences
Eukaryotes
NCLDVs
Figure S6

Supplement: Figure S6 — Bayesian phylogenetic tree of FEN (37 sequences, 304 positions). Detailed legend is the same as in Figure S1. (PPT) [file pone.0015530.s006.ppt]

## Slide 1
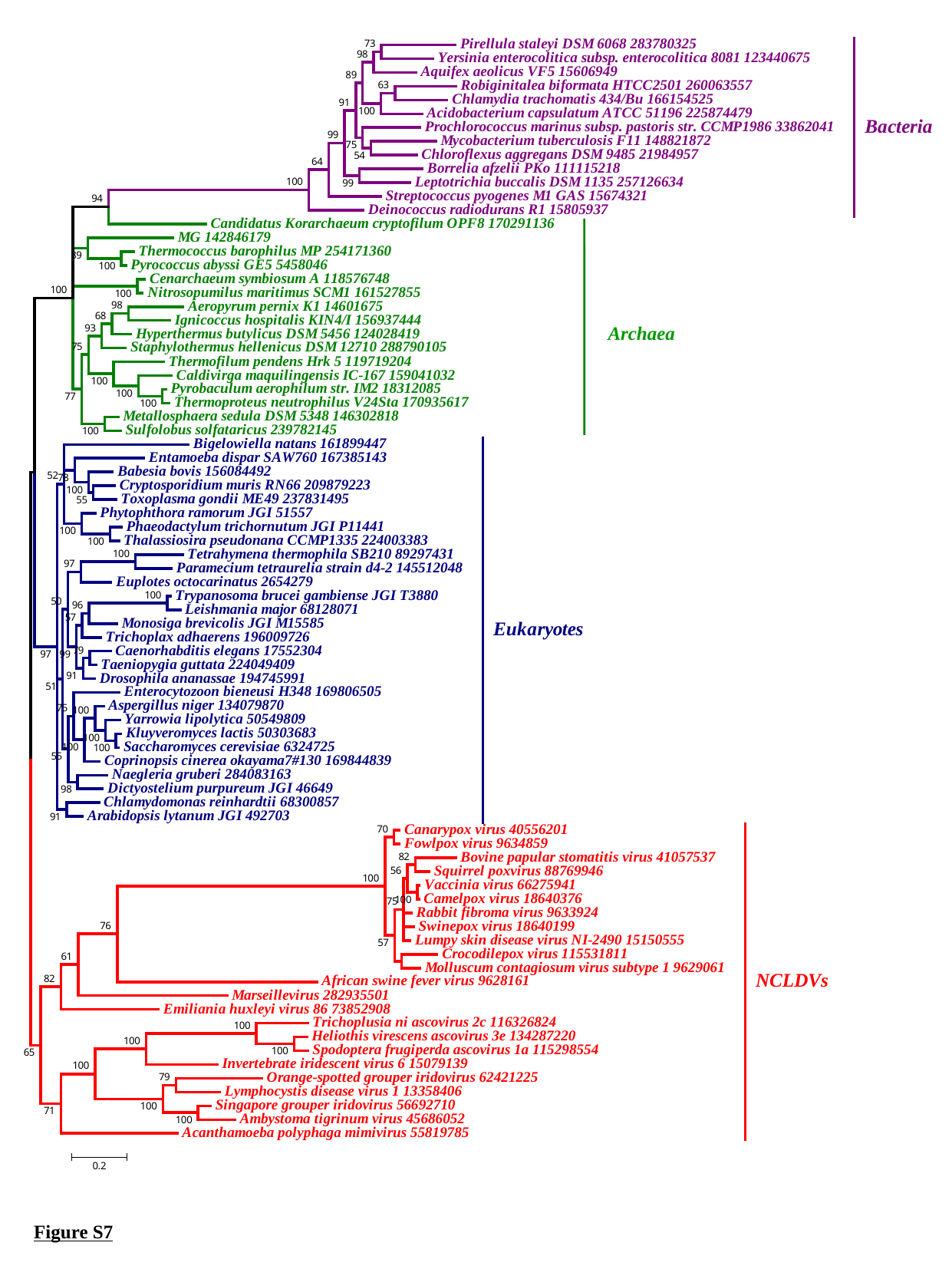

Archaea
Figure S7

Supplement: Figure S7 — Bayesian phylogenetic tree of RNAP II (80 sequences, 272 positions). Detailed legend is the same as in Figure S1. (PPT) [file pone.0015530.s007.ppt]

## Slide 1
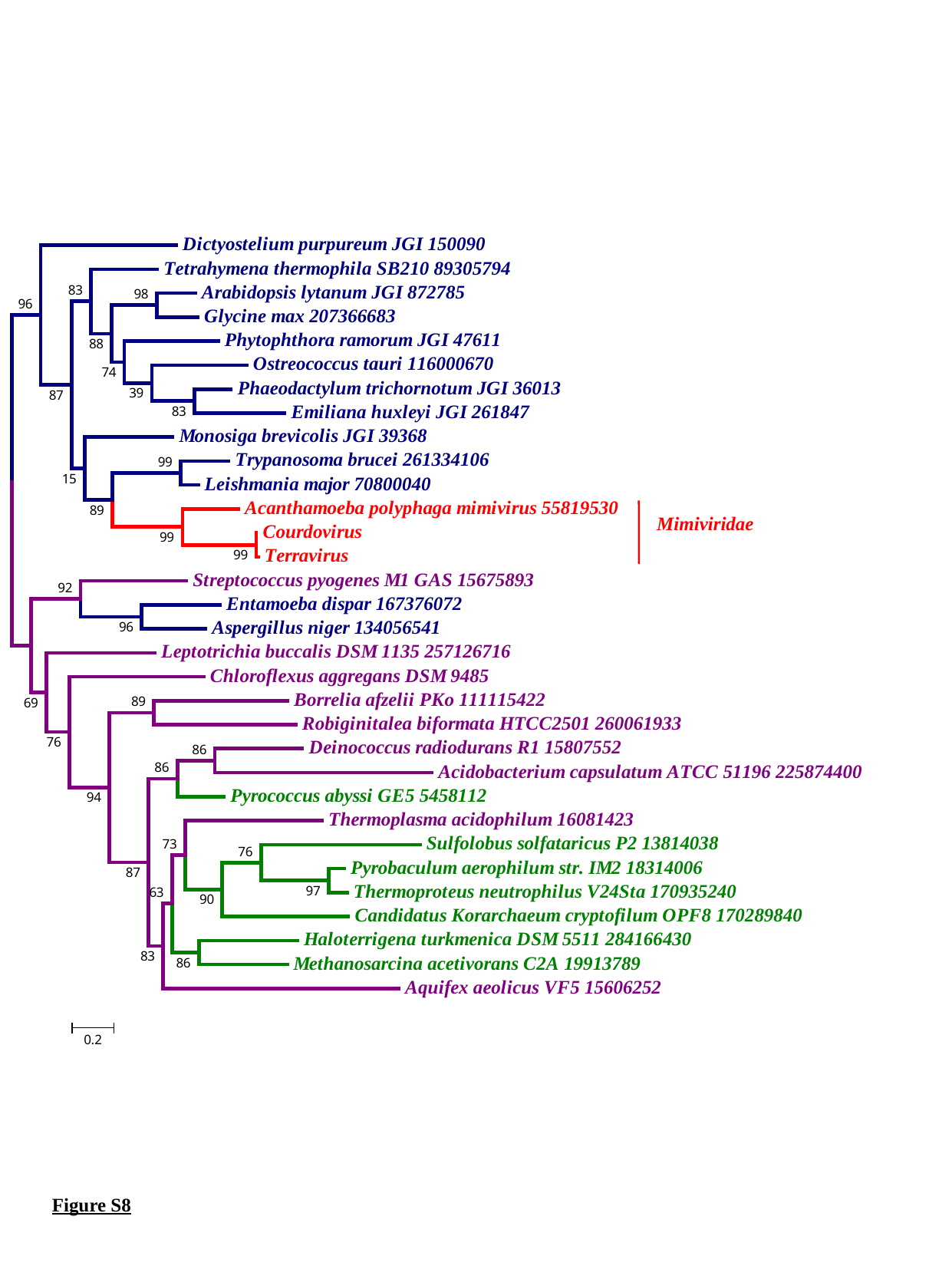

Mimiviridae
Figure S8

Supplement: Figure S8 — Maximum-likelihood phylogenetic tree of arginyl-tRNA synthetase (32 sequences, 123 positions). Detailed legend is the same as in Figure S1 except that numbers at nodes are SH-like local supports. (PPT) [file pone.0015530.s008.ppt]

## Slide 1
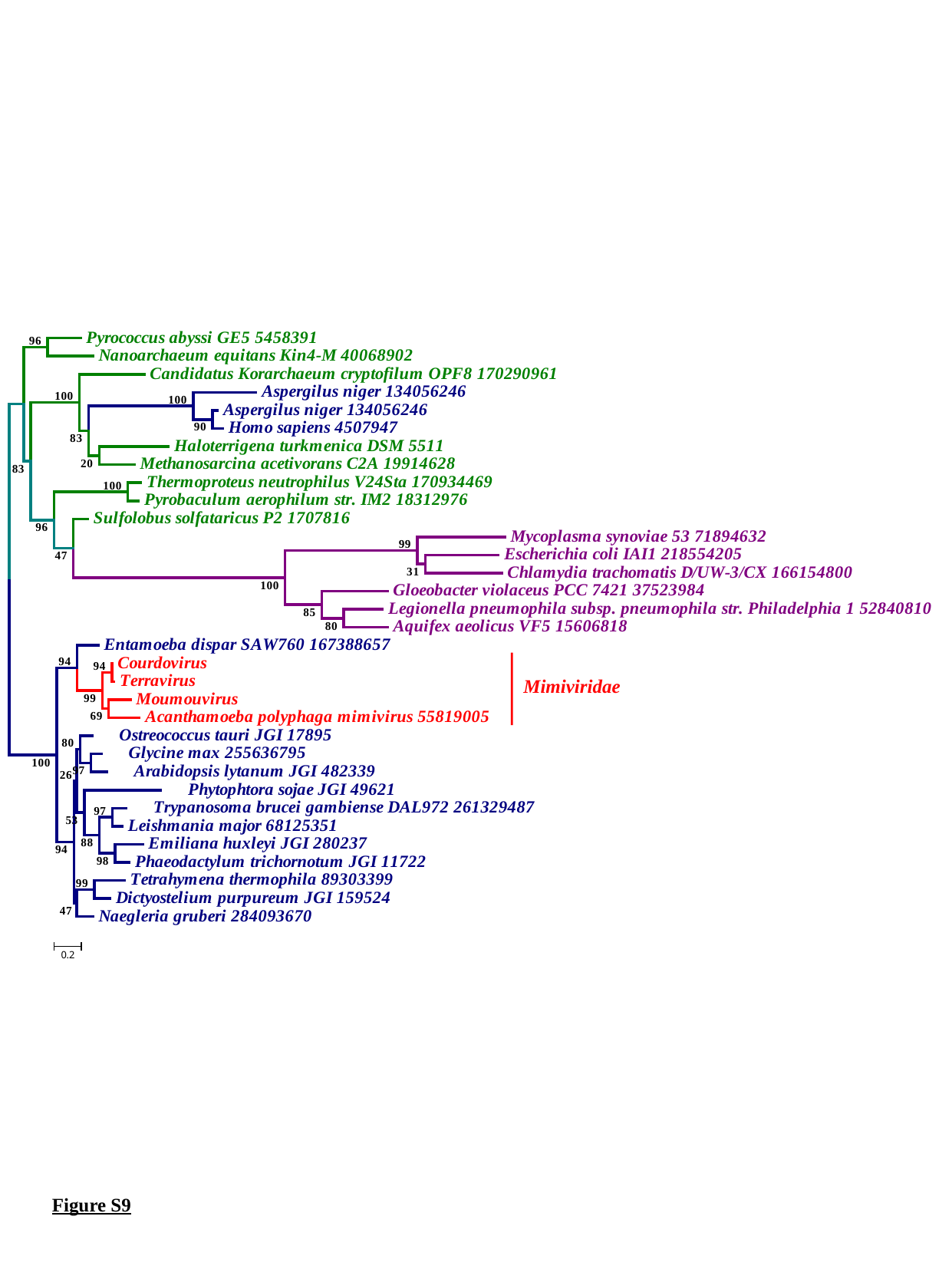

Mimiviridae
Figure S9

Supplement: Figure S9 — Maximum-likelihood phylogenetic tree of tyrosyl-tRNA synthetase (33 sequences, 201 positions). Detailed legend is the same as in Figure S1 except that numbers at nodes are SH-like local supports. (PPT) [file pone.0015530.s009.ppt]

## Slide 1
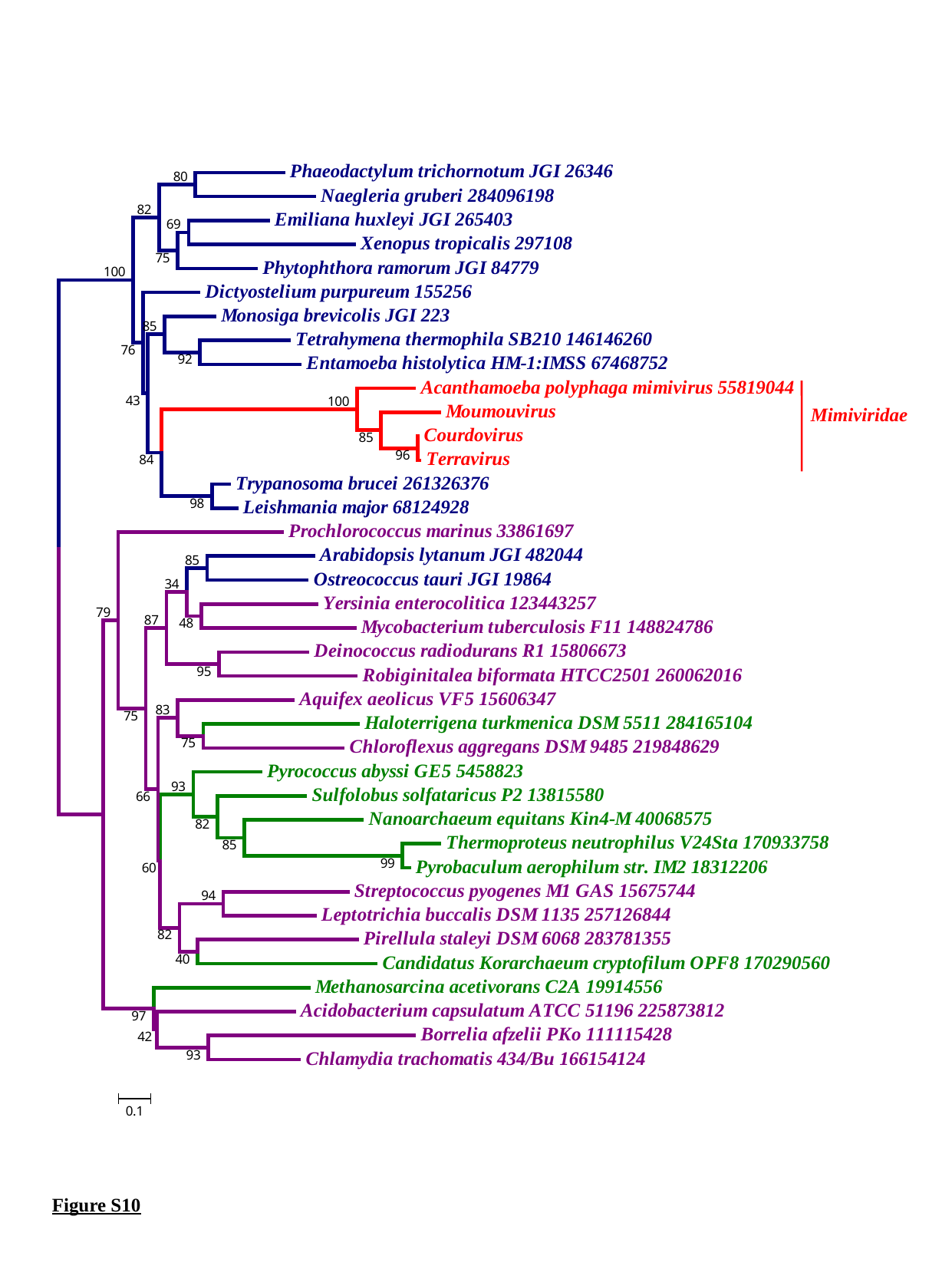

Mimiviridae
Figure S10

Supplement: Figure S10 — Maximum-likelihood phylogenetic tree of cysteine-tRNA synthetase (38 sequences, 156 positions). Detailed legend is the same as in Figure S1 except that numbers at nodes are SH-like local supports. (PPT) [file pone.0015530.s010.ppt]

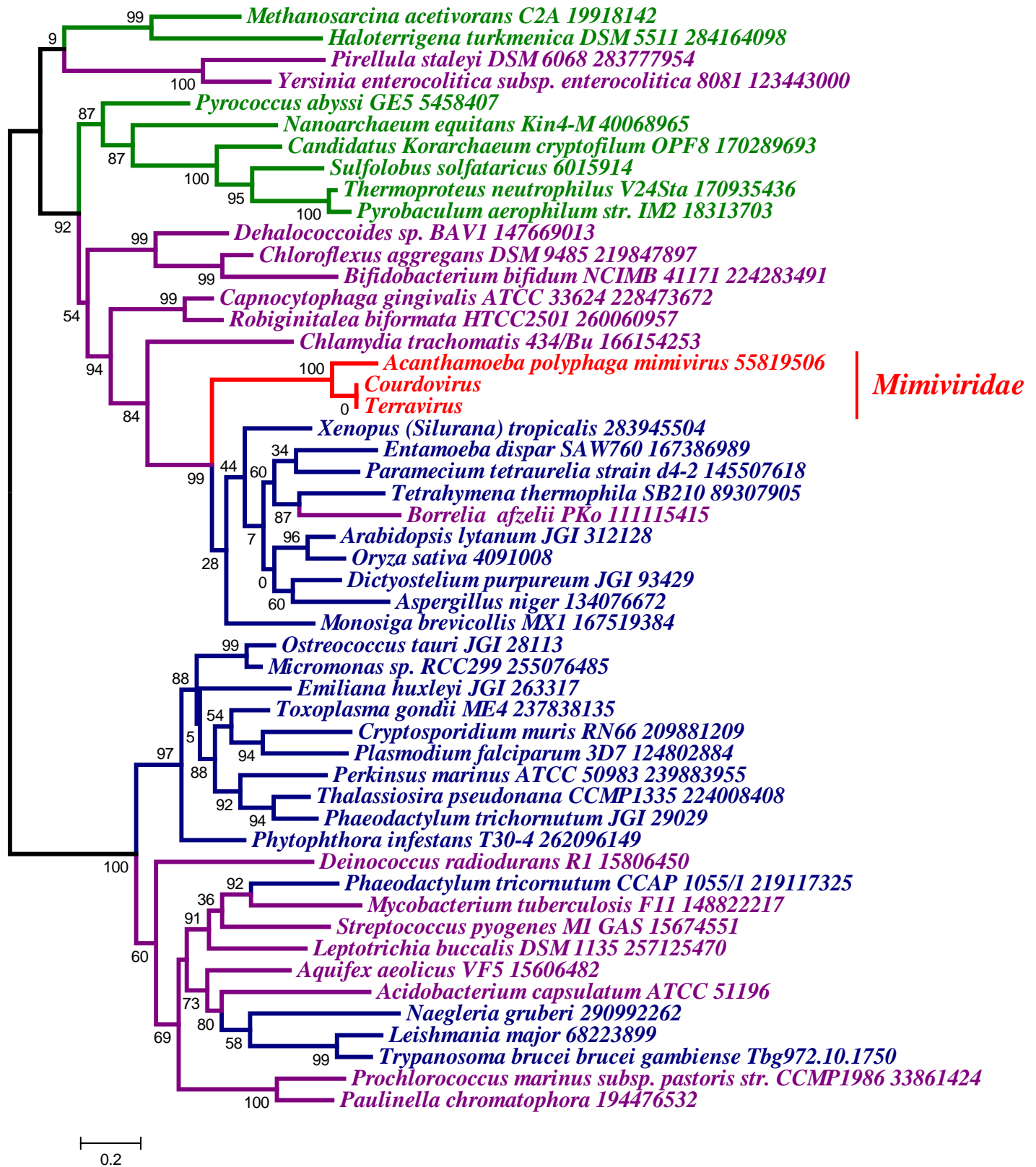

**Figure S11**

Supplement: Figure S11 — Maximum-likelihood phylogenetic tree of methyonyl-tRNA synthetase (51 sequences, 204 positions). Detailed legend is the same as in Figure S1 except that numbers at nodes are SH-like local supports. (PDF) [file pone.0015530.s011.pdf]
