# Supplementary material for: Phylogenetic and Phyletic Studies of Informational Genes in Genomes Highlight Existence of a 4th Domain of Life Including Giant Viruses
Source: PLoS One. 2010 Dec 2;5(12):e15530. doi: 10.1371/journal.pone.0015530 (PMC2996410; doi:10.1371/journal.pone.0015530)
Supplement: Figure S12 — Bayesian phylogenetic tree of EF-1 like phylogenetic tree (65 sequences, 157 positions). Detailed legend is the same as in Figure S1. (PPT) [file pone.0015530.s012.ppt]

## Slide 1
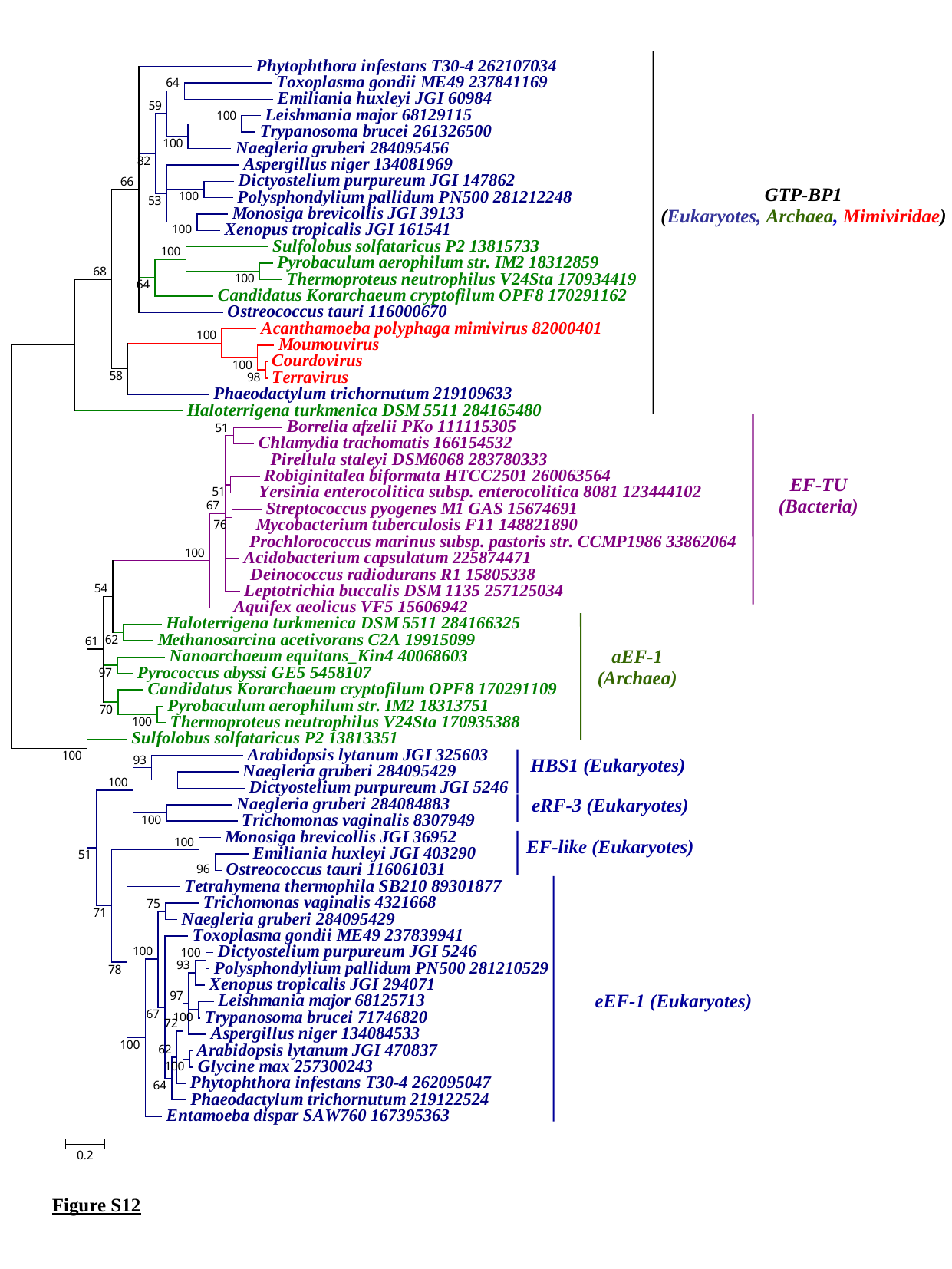

GTP-BP1
(Eukaryotes, Archaea, Mimiviridae)
EF-TU
(Bacteria)
aEF-1
(Archaea)
HBS1 (Eukaryotes)
eRF-3 (Eukaryotes)
EF-like (Eukaryotes)
eEF-1 (Eukaryotes)
Figure S12
